# Different Cardiovascular Responses to a Resistance Training Session in Hypertensive Women Receiving Propanolol Compared with Normotensive Controls

**DOI:** 10.1100/2012/913271

**Published:** 2012-05-03

**Authors:** Fabiano Moraes Miguel, Luis Alexandre Grings, Guilherme Borges Pereira, Richard Diego Leite, Amilton Vieira, Nuno Manuel Frade de Sousa, Roberto Simão, Jonato Prestes

**Affiliations:** ^1^Graduation Program in Exercise Physiology, Gama Filho University, São Paulo, SP, Brazil; ^2^Department of Physiological Sciences, Federal University of São Carlos, São Carlos, SP, Brazil; ^3^Physical Education Post-Graduation Program, Federal University of Rio de Janeiro, Rio de Janeiro, Brazil; ^4^Graduate Program on Physical Education and Health, Catholic University of Brasilia, 71966-700 Brasilia, DF, Brazil

## Abstract

The aim of the present study was to compare the responses of blood pressure, heart rate, and rate-pressure product of hypertensive women using beta-blockers with healthy controls during resistance exercise (by the end of the concentric phase of the contractions) and in the postexercise period (5 and 30 minutes after). Ten untrained normotensive women (N) and 10 mildly hypertensive females receiving 40 mg/day of propanolol (H) were selected. Three sets of 10 repetitions at 80% of 10 repetitions maximum with 30 s rest interval were performed on the leg press exercise. The H group exhibited lower systolic blood pressure after the second set compared with N. Heart rate and rate-pressure product were lower in H in all analyzed periods compared with N. Propanolol attenuates the cardiovascular response to a leg press resistance exercise in mildly hypertensive women.

## 1. Introduction

Resistance training (RT) is a noninvasive and nonpharmacological tool that improves specific health parameters and invokes benefits to both the cardiovascular and musculoskeletal systems. Health parameters such as muscle strength, flexibility, and body composition are intimately related with individual functional capacity and help to maintain quality of life by increasing the ability to maintain independent living with as one ages [1].

On the other hand, hypertension alone is a risk factor for several cardiovascular diseases [2], such as stroke, coronary heart disease, heart failure, peripheral arterial disease, and renal insufficiency [3]. Independently, the decrease in blood pressure (BP), heart rate (HR), and rate-pressure product (RPP) reduces the risk of vascular disease [3]. One elective strategy to control these cardiovascular variables is pharmacological therapy, for example, the use of beta-blockers, which are capable of antagonizing the sympathetic effects on the heart and consequently reduce HR and increase end-diastolic volume and stroke volume [4]. Beta-blockers are recommended for patients with heart failure, ischemic cardiomyopathy, arrhythmia, and hypertension [5].

RT is modality that has been shown to promote benefits on certain cardiovascular variables, both in sedentary and hypertensive individuals [1]. The cardioprotective effects of regular physical activity are decrease in systolic blood pressure (SBP) and diastolic blood pressure, postexercise hypotension, and reduced resting heart rate [1]. These effects occur either as a result of chronic adaptation or in acute responses of cardiovascular variables after a single exercise session [5].

Resistance training progression and adaptation relies on the manipulation of training variables such as volume, frequency, velocity of muscle contraction, exercise order, and the rest interval length between sets [6]. It has been shown that shorter rest intervals between sets (<1.5 minutes) promote superior hypertrophic and hormonal stimuli and modify to a higher amount the energetic metabolism [7, 8].

The magnitude of BP, HR, and RPP responses during RT are directly related to intensity, the number of repetitions and sets, and the rest interval [9]. Recently, Scher et al. [10] demonstrated that training volume exerted strong influences on cardiovascular response in elderly individuals during hypertension treatment. The results demonstrated that two passages in the circuit resistance training at 40% of one repetition maximum (1RM) was more effective in reducing SBP during a 24 h period than one passage. Previous research has shown a decrease in postexercise SBP after circuit RT (five exercises at 50% of one-maximum repetition) in normotensive and hypertensive women [11]. However, the cardiovascular response after multiple set RT with short rest interval between sets in hypertensive women using beta-blockers has not been investigated. This is particularly important since cardiovascular variables are modified to a greater extent after multiple sets compared with single set [3].

Considering the relevance of HR, BP, and RPP in the controlling and prescription of RT in hypertensive individuals, the objective of the present study was to compare the cardiovascular response during and after multiple set RT with short rest intervals in mildly hypertensive women receiving 40 mg/day of propanolol with normotensive women. Our initial hypothesis was that women on beta-blockers would present an attenuated cardiovascular response during and after a RT session compared with healthy controls.

## 2. Methods

### 2.1. Participants

Ten healthy women (N) and 10 mildly essential hypertensive women (H) receiving a daily oral morning dose of propanolol (40 mg/day) for at least six months before the intervention participated in the study. Before the drug treatment with propanolol, mildly hypertensive patients exhibited values of 140–159 mmHg for systolic blood pressure, and 90–99 mmHg for diastolic blood pressure. Hypertensive individuals were selected from the unit of hypertension treatment of Pelotas, Brazil. According to American College of Sports Medicine guidelines [12], participants were considered untrained, having had no regular practice of strength training in the six previous months prior to participating in the study. Training background and habitual physical activity was assessed by a personal interview. As a part of the initial trial, participants completed a health evaluation questionnaire that was analyzed by a physician specialist in Sports Medicine. None of the participants presented other cardiovascular diseases, osteomioarticular or target organ damage that might influence the performance of the proposed exercises. Cardiovascular risk factors such as diabetes and hypercholesterolemia were excluded in both groups. Anthropometric characteristics of the participants are presented in Table 1. All participants signed an informed consent document, and the experimental protocol was approved by the Research Ethics Committee of the Institution. All procedures were conducted in accordance with the Declaration of Helsinki (1964).

Participants did not ingest caffeine or alcohol during the 24-hour period prior to any of the testing protocols and did not perform any rigorous physical activity during the 48 hours prior to testing. All trials were performed at the same period of the day to avoid any influence of circadian rhythm on cardiovascular variables.

## 3. Experimental Protocol

All subjects underwent an anthropometric (body mass, height, and skinfolds thickness) and cardiac evaluation, including a resting 12-lead electrocardiogram. Following the resting electrocardiogram, all participants performed three orientation sessions to become familiar with the ten repetition maximal (10RM) test, with intervals between each session ranging from 48 to 72 h. Familiarization sessions were performed on the 45° leg press machine (Cybex International, Medway, MA) using a training load corresponding to 15RM. The training load corresponding to 15RM was used to indirectly determine the load for the first 10RM test trial. The 10RM tests were then performed in two nonconsecutive days. The first 10RM test was performed, and, then after 48 h, the second 10RM test was repeated to determine test-retest reliability. The RT session was performed 72 h after the second 10RM test.

### 3.1. Ten Repetitions Maximum Testing (10RM)

A light warmup with ten submaximal repetitions was instructed with a two-minute rest before the initiation of the tests. To obtain a reliable 10RM load, data was assessed during two nonconsecutive days in the 45° leg press (LP) (Cybex International, Medway, MA). The 10RM load was determined by using the maximum weight that could be lifted for ten consecutive repetitions at a constant velocity of 3 seconds per repetition (1.5 s in concentric and 1.5 s in the eccentric phase). If the subject could not accomplish the 10RM in the first attempt, the weight was adjusted by 4–10 kg. Each subject performed a maximum of five 10RM attempts with 5-minute rest intervals between attempts. To minimize the error during tests, the following strategies were adopted according to Simão et al. [13]: (a) standardized instructions concerning the testing procedure were given to the participants before the test; (b) participants received standardized instructions on exercise technique; (c) verbal encouragement was provided during the testing procedure; (d) the mass of the leg press sled was considered. A paired student *t*-test did not show significant differences between the 10RM tests (intraclass correlation coefficient *r* = 0.93, *P* ≤ 0.05). The heaviest load achieved was considered the 10RM.

### 3.2. Resistance Training Session

Prior to the RT session, volunteers rested quietly in a seated position for 20 minutes to facilitate baseline measurements of systolic blood pressure (SBP), diastolic blood pressure (DBP), and heart rate (HR). Before the session, a specific warmup with 1 set of 10 repetitions at 50% of 10RM was performed. Subsequently, the RT was initiated, and volunteers performed three sets, of 10 repetitions at 80% of 10RM in the 45° leg press, with 30 s rest interval between sets. No pauses between concentric and eccentric actions were allowed, and investigators were present to assure that standardized exercise technique was maintained. Volunteers were instructed to avoid the Valsalva maneuver during the RT session [1] in order to minimize potentially abrupt modifications in the cardiovascular response with exercise. A preliminary orientation to establish appropriate weight loads and instruct the participant on proper lifting techniques, range of motion of the leg press exercise, and correct breathing patterns were taken to avoid Valsalva maneuver. During sets BP and HR were measured between the last two sets by the end of the concentric phase of the contractions and in the postexercise period (5 and 30 minutes after). All BP measurements (pre- and postsession) were obtained using the auscultatory method with appropriate cuffs fitted according to the size of the upper arm of each participant. The procedures for BP measurement were in accordance with guidelines from the American Heart Association [14]. This method has been validated by the Association for the Advancement of Medical Instrumentation and by the British Hypertension Society. In spite of the indirect blood pressure have been used widely, the protocol can present bias. For this reason, during BP and HR monitoring, participants remained in a seated position in a temperature controlled, quiet room (23°C). Heart rate was measured using telemetry (Polar, MZ1, Finland). Rate-pressure product was calculated by multiplying SBP by HR.

### 3.3. Statistical Analysis

 The data are presented as mean ± standard deviation of the mean. The sample size was calculated considering 1.8 mmHg as the minimum difference in the resting SBP value between the groups, the residual standard deviation was 0.75, and the statistical power was 0.80. All variables presented normal distribution and homocedasticity, and a 2 × 6 ANOVA with two independent variables (group-normotensive versus hypertensive women, and time-six different time periods) was computed. Bonferroni's posthoc test was applied in the event of a significant at (*P* < 0.05) *F* ratio. The calculation of the effect size (ES) for the cardiovascular variables was performed according to the classification proposed by Rhea [15]. Statistical analysis was performed using Statistics 6.0 for Windows (Statsoft, Tulsa, OK, USA) with a critical level accepted *P* < 0.05.

## 4. Results

 There were no statistically significant differences in anthropometric variables between groups (Table 1). Mean values for HR and BP before, during, and after the RT session in normotensive women (N) are presented in Table 2. All cardiovascular variables, except for DBP, exhibited a significant increase after 3 sets of RT compared with resting values. While DBP tended to rise with increasing sets of RT exercise, no significant difference was observed at any point when compared to baseline or recovery values. RPP presented higher values after sets 2 and 3 compared with set 1 (*P* = 0.02).

 There were no significant changes in HR during the 3 sets of 45° leg press when only exercise conditions were compared (i.e., HR increased with the first set of RT and remained elevated at the same extent with successive sets). SBP, HR, and RPP were lower during the recovery period compared with the values found during the 3 sets of RT (*P* = 0.001). Moreover, SBP dropped below resting 30 min after exercise.

Mean values for HR and BP before, during and after the RT session in the hypertensive group (H) are presented in Table 3. There was a significant increase in SBP, HR, and RPP during the 3 sets of RT compared with resting baseline values (*P* < 0.001). When only the exercise conditions were compared, no differences were observed among RT sets for SBP, DBP, HR and RPP responses. After five minutes of recovery, SBP and RPP exhibited lower values compared with resting and RT conditions (*P* < 0.05). Lower HR values were found after five and 30 minutes of recovery compared with the RT session, but not compared with resting values.

**Figure 1:**
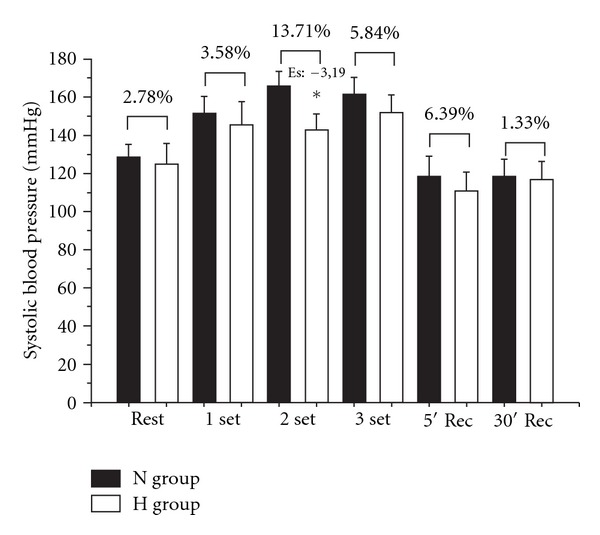
Comparison of systolic blood pressure (SBP) between N (normotense control) and H (hypertensive using propanolol) before, during, and after a resistance exercise session. Mean ± standard deviation of the mean. *Difference between groups (*P* ≤ 0.05).

**Figure 2:**
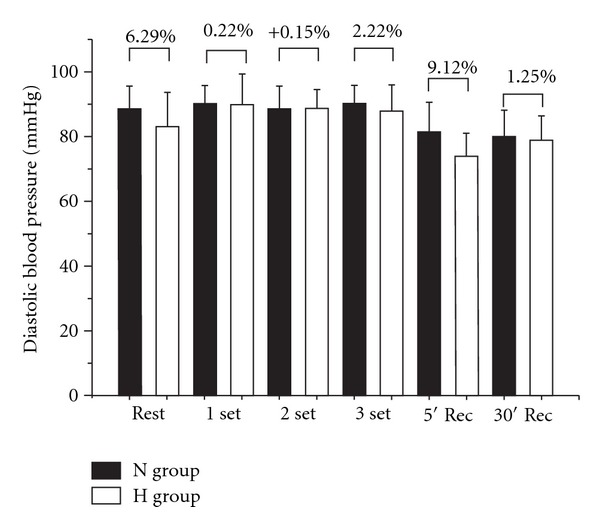
Comparison of diastolic blood pressure (DBP) between N (normotense control) and H (hypertensive using propanolol) before, during, and after a resistance exercise session. Mean ± standard deviation of the mean. *Difference between groups (*P* ≤ 0.05).

**Figure 3:**
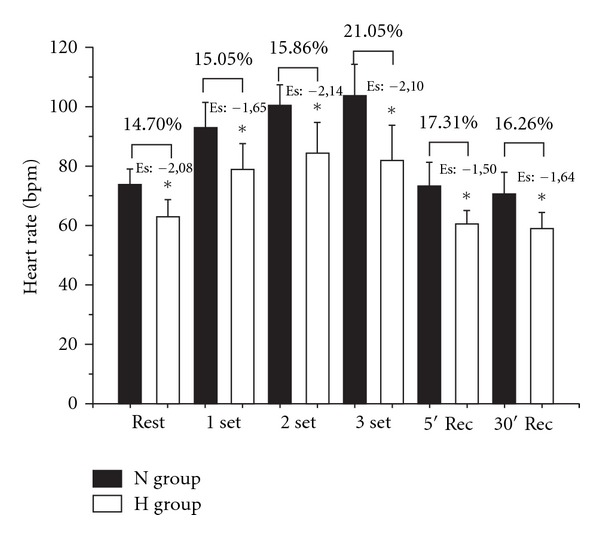
Comparison of heart rate (HR) between N (normotensive control) and H (hypertensive using propanolol) before, during, and after a resistance exercise session. Mean ± standard deviation of the mean. Es: effect size. *Difference between groups (*P* ≤ 0.05).

**Figure 4:**
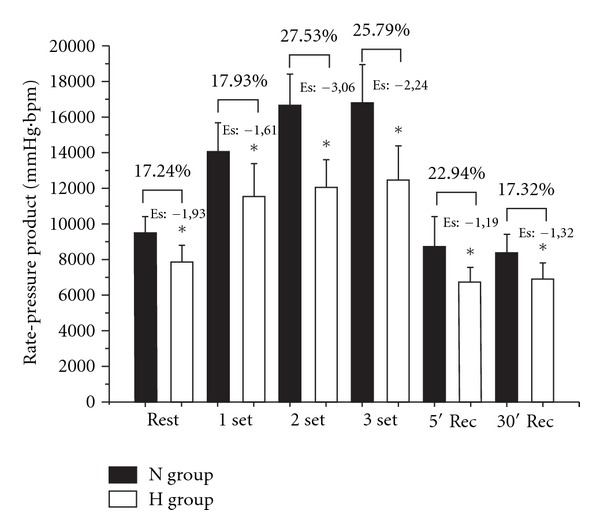
Comparison of rate-product pressure (RPP) between N (normotensive control) and H (hypertensive using propanolol) before, during, and after a resistance exercise session. Mean ± standard deviation of the mean. Es: effect size. *Difference between groups (*P* ≤ 0.05).

H group exhibited a lower SBP during set 2 compared with N (Figure 1). No differences in DBP were noted between N and H in any condition (Figure 2). HR and RPP presented lower values for H in all periods as compared with N group (Figures 3 and 4, resp.). 

## 5. Discussion

 The present study reveals the important clinical role of 40 mg/day of propanolol in attenuating the cardiovascular responses to a RT session in mildly hypertensive individuals. In addition, these results have direct implications for RT prescription, highlighting the safety of this type of exercise for mildly hypertensive patients treated with propanolol. During three sets of RT, hypertensive women treated with propanolol exhibited lower values of HR and consequently of RPP compared with normotensive women. Additionally, propanolol modulated cardiovascular variables during resting and in the recovery period of a RT session. This indicates that beta-blockers, such as propanolol, are cardioprotective at rest and during a RT session in hypertensive individuals, who have a higher risk of developing coronary heart disease, cardiac ischemia, and acute myocardial infarction [5].

While the intra-arterial method is considered the gold-standard for determining blood pressure [16], we chose to utilize the auscultatory method in the present study. Wiecek et al. [16] found that the indirect (auscultatory) method underestimates pressure values by 15% during sets and by 30% immediately after exercise compared to intra-arterial values. However, the correlation between these methods is significant. In addition, the reliability of the indirect method has been confirmed during moderate/high intensity exercise [17]. Finally, due to the invasive nature of the procedure, the intra-arterial method imposes an additional stress on the heart, which we wished to avoid particularly in the hypertensive women who participated. Therefore, the indirect method to obtain BP measures was employed during the current investigation. 

In the present study, normotensive individuals exhibited normal cardiovascular responses in the analyzed variables. The increases in SBP, HR, and RPP during RT were of a similar magnitude compared to previous studies which utilized low, moderate, and high intensity RT [16, 18, 19]. An acute RT session is capable of inducing postexercise hypotension (PEH) in healthy and hypertensive individuals [20], and this effect occurs in both SBP and DBP measurements [21]. In the present study, individuals using beta-blockers exhibited PEH only in the 5th minute of recovery. Fisher [21] evaluated PEH after a circuit RT every 10 min for 60 min in normotensive and hypertensive individuals and found that both groups presented PEH.

Furthermore, it should be considered that the manipulation of training variables, such as number of sets and repetitions, intensity, and the rest interval between sets, can induce different cardiovascular responses. For example, Nery et al. [22] showed that the increase in SBP during resistance exercise was greater in hypertensive than in normotensive individuals. Additionally, they found that, in hypertensive and normotensive subjects alike, low-intensity resistance exercise (3 sets in the knee extension at 40% of 1RM), when performed to concentric failure, induced a higher increase in SBP compared with three sets at 80% of 1RM.

In the study of Nery et al. [22], there was an exacerbated BP response even when individuals performed 6–8 maximal repetitions at 80% of 1RM. On the other hand, in the present study, training loads were adjusted to avoid excessive cardiovascular stress by using 80% of 10 RM, which characterizes a submaximal training (not to failure). However, our results should be interpreted with caution as we had a reduced number of participants.

In the resting condition, individuals using propanolol presented a lower HR and this may be attributed to the attenuating chronotropic and inotropic effect of beta-blockers [23, 24]. The increase in HR during RT sets observed in normotensive individuals is associated with a stimulus on mechano- and metaboreceptors in skeletal muscle, promoting a sympathetic discharge on the cardiovascular system [25], and to a direct effect of plasma adrenalin on the heart. Additionally, when sets were compared between groups, hypertensive individuals treated with propanolol presented a ~20% lower HR value, corroborating the findings of a previous study that showed a similar blunted HR response during dynamic exercise in beta-blocker users [23].

When compared with other antihypertensive medications, beta-blockers present an optimal effect in reducing BP, HR, and myocardial contractility, which are the three main determinants of oxygen consumption by cardiac muscle [3, 19]. These attributes are important in the treatment of hypertension, arrhythmia, and heart failure. The cardioprotective effects of propanolol can also be observed on RPP during RT, which is a relevant issue for exercise prescription and safety. Additionally, for similar a intensity, and volume, RT promotes lower absolute values of RPP compared with aerobic exercise [26]. In addition to cardioprotection, RT increases muscle endurance and strength, functional capacity, independence, along with acute and chronic adaptations that improve cardiac function [1].

In conclusion, 40 mg/day of propanolol attenuates the cardiovascular response to RT, mainly in HR, SBP, and RPP. Additional studies involving other RT variables such as: exercises for other muscle groups, single- and multiple-joint exercises, different intensity and volume are necessary to improve the understanding of the interplay between RT and beta-blocker use. The prescription of RT with different doses of beta-blockers should also be investigated.

## Figures and Tables

**Table 1:** Anthropometric characteristics of the groups.

Variables	N (*n* = 10)	H (*n* = 10)
Age (y)	52.1 ± 10.7	56.5 ± 9.6
Body mass (kg)	71.7 ± 4.7	73.4 ± 17.4
Height (cm)	163.3 ± 5.2	156.3 ± 5.2
Body fat percentage (%)	32.9 ± 6.1	36.4 ± 5.7

Mean ± standard deviation of the mean of N: normotensive control women, H: hypertensive women using propanolol.

**Table 2:** Cardiovascular responses to an acute resistance training session in the normotensive control women (N Group).

Period	SBP	DBP	HR	RPP
Resting	129 ± 6.9^a^	89 ± 6.9	74 ± 5.3^a^	9.500 ± 908.7^a^
Set 1	151 ± 9.0	90 ± 5.8	93 ± 8.5	14.100 ± 1619.0^b^
Set 2	166 ± 7.2	100 ± 6.9	100 ± 7.0	16.100 ± 1342.4
Set 3	171 ± 6.7	103 ± 10.5	104 ± 10.5	16.900 ± 2007.8
5′ after	119 ± 10.7^a^	81 ± 9.0	73 ± 8.0^a^	8.700 ± 1677.8^a^
30′ after	119 ± 9.0*	80 ± 8.2	71 ± 7.3^a^	8.300 ± 1064.5^a^

Mean ± standard deviation of the mean. *Different from resting, set 1, 2, and 3; ^a^different from set 1, 2, and 3; ^b^different from set 2 and 3, *P* ≤ 0.05. SBP: systolic blood pressure, DBP: diastolic blood pressure, HR: heart rate, RPP: rate-pressure product.

**Table 3:** Cardiovascular responses to an acute resistance training session in the hypertensive women using propanolol (H Group).

Period	SBP	DBP	HR	RPP
Resting	125 ± 10.8^a^	83 ± 10.6	63 ± 5.8^a^	7.800 ± 935.4^a^
Set 1	146 ± 11.7	90 ± 9.4	79 ± 8.7	11.500 ± 1817.6
Set 2	143 ± 8.2	89 ± 5.7	85 ± 10.4	12.000 ± 1583.2
Set 3	152 ± 9.2	88 ± 7.9	82 ± 11.8	12.400 ± 1963.5
5′ after	111 ± 9.9*	74 ± 7.0^a^	61 ± 4.4^a^	6.700 ± 821.5*
30′ after	117 ± 9.5^a^	79 ± 7.4^a^	59 ± 5.3^a^	6.900 ± 875.3*

Mean ± standard deviation of the mean. *Different from resting, set 1, 2, and 3; ^a^different from set 1, 2, and 3, *P* ≤ 0.05. SBP: systolic blood pressure, DBP: diastolic blood pressure, HR: heart rate, RPP: rate-pressure product.
